# Incidence of Cytomegalovirus disease and viral replication kinetics in seropositive liver transplant recipients managed under preemptive therapy in a tertiary-care center in Mexico City: a retrospective cohort study

**DOI:** 10.1186/s12879-022-07123-w

**Published:** 2022-02-14

**Authors:** Oscar A. Fernández-García, Ignacio García-Juárez, Pablo Francisco Belaunzarán-Zamudio, Mario Vilatoba, Andrea Wisniowski-Yáñez, Jacobo Salomón-Ávila, Miriam Bobadilla-del-Valle, José Sifuentes-Osornio, Jennifer M. Cuellar-Rodríguez

**Affiliations:** 1 Infectious Diseases Department, Instituto Nacional de Ciencias Médicas y Nutrición Salvador Zubirán, Vasco de Quiroga 15, Belisario Domínguez Sección XVI, Tlalpan, 14080 Mexico City, Mexico; 2 Gastroenterology Department, Instituto Nacional de Ciencias Médicas y Nutrición Salvador Zubirán, Vasco de Quiroga 15, Belisario Domínguez Sección XVI, Tlalpan, 14080 Mexico City, Mexico; 3 Transplant Department, Instituto Nacional de Ciencias Médicas y Nutrición Salvador Zubirán, Vasco de Quiroga 15, Belisario Domínguez Sección XVI, Tlalpan, 14080 Mexico City, Mexico; 4 Department of Medicine, Instituto Nacional de Ciencias Médicas y Nutrición Salvador Zubirán, Vasco de Quiroga 15, Belisario Domínguez Sección XVI, Tlalpan, 14080 Mexico City, Mexico; 5 Independient Researcher, Teresa 703, Jardines del Santuario, 31206 Chihuahua, Mexico; 6 Clinical Microbiology Laboratory, Instituto Nacional de Ciencias Médicas y Nutrición Salvador Zubirán, Vasco de Quiroga 15, Belisario Domínguez Sección XVI, Tlalpan, 14080 Mexico City, Mexico

**Keywords:** Cytomegalovirus, Preemptive therapy, DNAemia, Viremia, Replication

## Abstract

**Background:**

In the absence of an adequate prevention strategy, up to 20% of CMV IgG+ liver transplant recipients (LTR) will develop CMV disease. Despite improved reporting in CMV-DNAemia, there is no consensus as to what the ideal CMV-DNAemia cutoff for a successful preemptive strategy is. Each transplant centre establishes their own threshold. We aimed to determine the effectiveness of our preventive strategy in CMV IgG+ LTR, and evaluate CMV replication kinetics.

**Methods:**

In this retrospective study we determined the incidence of CMV disease in the first 6 months following transplantation in CMV seropositive LTR in a tertiary-care centre in Mexico. Secondary outcomes were determining the number of patients who required preemptive therapy (treatment cutoff ≥ 4000 UI/ml), adherence to the centre’s prevention protocol and calculation of viral replication kinetics.

**Results:**

One-hundred and twenty-four patients met inclusion criteria. Four patients (3.2%) developed CMV disease. Ninety-six (85%) had detectable DNAemia and 25 (22%) asymptomatic patients received preemptive therapy, none of them developed CMV disease. The highest viral loads were observed on the second posttransplant month. The number of viral load measurements decreased over time. Patients with DNAemia ≥ 4000 UI/ml had a faster viral load growth rate, shorter viral load duplication time, and higher basic reproductive number. Viral load growth rate and autoimmune hepatitis were associated with development of DNAemia ≥ 4000 UI/ml.

**Conclusion:**

Cytomegalovirus disease occurred in 3.2% of the study subjects. Preemptive therapy using a threshold of CMV ≥ 4000 UI/ml was effective in reducing the incidence of end-organ disease. The viral replication parameters described in this population highlight the importance of frequent monitoring, a challenging feat for transplant programs in low- and middle-income countries.

**Supplementary Information:**

The online version contains supplementary material available at 10.1186/s12879-022-07123-w.

## Background

Cytomegalovirus (CMV) is a major pathogen affecting solid organ transplant (SOT) recipients. It can lead to organ damage and is associated with organ rejection [1]. Up to 20% of CMV IgG-positive liver transplant recipients develop disease in the absence of a prevention strategy. Periodical measurement of CMV DNAemia and initiation of treatment once a threshold is reached is known as preemptive therapy [2].

Despite standardization by the World Health Organization (WHO) in 2010 there is no universal DNAemia threshold at which therapy should be initiated. Results vary depending on whether it is measured in plasma or whole blood (whole blood yields consistently higher viral loads) [2]. Transplant centres establish and monitor their own criteria to initiate antiviral therapy in SOT recipients.

Patients who develop disease present higher maximum viral loads and faster replication. Viral load growth rate is the increase in viral load per unit of time. Viral load doubling time is another expression of the velocity of viral replication. The basic reproductive number is a parameter that expresses the number of new infections derived from a single infected cell when target cells are unlimited [3]. In seropositive recipients, viral load doubling time has been calculated to be 2.1–2.6 days and the median basic reproductive number has been determined to be 1.48 [4]. As expected, shorter doubling time and higher basic reproductive number have been reported in seronegative recipients of seropositive donors (D+/R−), 1.5 days and 2.02, respectively [4, 5].

## Methods

### Study design and outcomes

This was a retrospective cohort study of CMV IgG-positive adult liver transplant recipients managed under preemptive therapy for prevention of CMV disease. The study included CMV IgG-positive recipients of deceased-donor liver grafts from February 2017 to December 2019 in a tertiary-care centre located in Mexico City. The study was approved by the Ethics Committee of the Instituto Nacional de Ciencias Médicas y Nutrición Salvador Zubirán. The committee waived the need for informed consent. Data were retrieved from the hospital’s electronic health record.

The primary objective of the study was to calculate the cumulative incidence of CMV disease in the first 6 posttransplant months. Secondary objectives were to determine the number of patients who received preemptive therapy. We estimated adherence to the centre’s preemptive therapy protocol. We compared viral replication parameters between patients who did and who did not reach the centre’s preemptive therapy threshold (4000 UI/ml). We performed bivariate and multivariate analyses to examine variables associated with development of DNAemia ≥ 4000 UI/ml.

We recorded data from the day of the transplant procedure to posttransplant month 6. Data were censored at the time of the event if death, retransplantation or rejection with administration of high dose methylprednisolone occurred within the study period.

### Study criteria and diagnosis

Liver transplant recipients aged 18 or more and positive for CMV IgG before transplant were included. Patients receiving a second (or ulterior) liver graft, anti-thymocyte globulin induction or antiviral prophylaxis with valganciclovir were excluded.

All the recipients received induction therapy with methylprednisolone and basiliximab. Prednisone, tacrolimus, and mycophenolate mofetil were used for maintenance therapy. All recipients had CMV IgG status determined as part of the pretransplant evaluation. Due to the high prevalence of CMV infection in developing countries deceased donor CMV status is not mandatorily determined in the study centre. Donors are assumed positive for the purposes of recipient management. The centre’s CMV disease prevention protocol for CMV IgG-positive recipients recommends measuring CMV viral load starting the 1st week after transplant, on a weekly basis for the first three posttransplant months and every 2 weeks from months 4 to 6. The centre’s microbiology laboratory performs the VL load determination 2–3 times weekly, positive results are communicated to the infectious disease service immediately. Antiviral therapy with valganciclovir 900 mg twice daily (or ganciclovir 5 mg/kg twice daily) is initiated if a viral load ≥ 4000 UI/ml is documented. Outpatients are contacted to start therapy. The threshold was based on a derivation-validation study performed by Martin-Gandul et al. [5] Treatment is continued until 2 consecutive negative viral loads are observed. Patients who develop neutropenia are started on G-CSF, antiviral therapy is not stopped.

Except for retinitis, we defined CMV disease as the presence of histological evidence of CMV infection in tissue biopsy [6]. Clinical events deemed by the treating physician to be CMV disease and treated empirically as such were also considered CMV disease.

### Viral load measurement

Viral load was measured by real-time polymerase chain reaction in whole blood with the Elitech InGenious® (Elitech Group, Svizzera, Torino, Italy) instrument using the CMV Elite MGB Kit® according to the manufacturer’s instructions. The amplified region corresponds to exon 4 of the CMV major immediate early antigen (MIEA, HCMV UL123). The assay’s lower limit of detection is 109 UI/ml (71–239).

### Viral replication kinetics

We calculated the viral load growth rate (r), doubling time (Td) and basic reproductive number (R_0_) using the equations derived by Emery et al. [3, 4] Growth rate was calculated as r = lnVL2 − lnVL1/time. VL2 was the highest viral load recorded for each patient, VL1 was the viral load preceding VL2, time was expressed in days. Doubling time was calculated as Td = ln2/r. Basic reproductive number was calculated as R_0_ = 1 + (r/δ). δ is a constant representing the infected cells death rate and corresponds to 0.69 cells/day, we assumed no time delay between infection of subsequent cells. Replication kinetics were only calculated for patients who had a time between VL1 and VL2 ≤ 14 days.

### Statistical analysis

CMV viral loads were logarithmically expressed to ensure normal distribution. A mixed linear parametric model was used to determine if there were differences in viral load over time. Viral load means for months 1, 2 and 3 were compared using contrasts. Wilcoxon’s rank sum test was used to compare viral replication parameters between patients who developed DNAemia ≥ 4000 UI/ml and those who did not. Bivariate logistic regression was performed to explore variables associated with DNAemia ≥ 4000 UI/ml. Multivariate logistic regression was performed incorporating variables with p < 0.2 in bivariate analysis and variables known to be associated with posttransplant non-viral infections. A p value of ≤ 0.05 was considered as statistically significant. Data analyses were performed on STATA version 14.1, R version 3.4.0 and R studio version 1.0.143.

## Results

During the study period 144 patients had a liver transplant performed, 123 patients met inclusion criteria. Of the 21 excluded patients 14 had negative CMV IgG, 6 received a second graft and 1 received anti-thymocyte-globulin induction. Characteristics of included patients are summarized in Table 1. CMV IgG status was determined in 88 donors, of which 76 (86%) were IgG positive.

**Table 1:** Characteristics of the study population

Characteristic	N = 123
Women	64 (52%)
Age	52 years (18–69)
MELD	16 (6–40)
Child–Pugh A	9 (7%)
Child–Pugh B	44 (35%)
Child–Pugh C	64 (52%)
Hepatocellular carcinoma	28 (23%)
Hepatitis C virus	26 (21%)
Cryptogenic cirrhosis	26 (21%)
Primary biliary cholangitis	12 (9.5%)
Autoimmune hepatitis/primary biliary cholangitis overlap	8 (6.5%)
Autoimmune hepatitis	8 (6.5%)
Alcohol	8 (6.5%)
Benign bile duct lesions	8 (6.5%)
Non-alcoholic steatohepatitis	7 (5.6%)
Primary sclerosing cholangitis	6 (4.8%)
Other*	14 (11.3%)

*HCC* hepatocellular carcinoma

*Other causes included inborn errors of immunity, tumors, acute liver failure and drug-induced liver injury. These subjects did not have the Child–Pugh score calculated as they did not have cirrhosis

During the follow-up period 12 (10%) patients had acute graft rejection documented by biopsy, 5 (4%) required treatment with high-dose steroids. One (0.8%) patient required a second transplant due to early surgical complications.

Fourteen (11%) patients died. No death was directly attributed to CMV. No subjects were lost to follow up.

Regarding the primary outcome, four patients (3.2%, CI 1.2–8) developed CMV disease, two of them met the definition of proven disease with histopathological findings of CMV infection. The first one corresponded to CMV graft hepatitis associated with acute cellular rejection 2 weeks after transplantation. This patient had a negative blood CMV viral load at the time of diagnosis. The other patient developed CMV esophagitis with a rapid increase in viral load in 1 week; from less than 4000 UI/ml to 55,119 UI/ml at the time of endoscopy. Two patients were considered to have CMV disease by their treating physicians. One had bilateral interstitial pneumonia with a CMV viral load of 152,786 UI/ml. This subject was contacted when a viral load ≥ 4000 UI/ml was detected, he did not return to the hospital until several weeks later. The last patient had mild elevation of aminotransferases and a CMV viral load of 1773 UI/ml. The patient received antiviral therapy. Mild liver enzyme elevation persisted until optimization of tacrolimus levels.

One-hundred and thirteen subjects had viral load measured at least once before censoring. Ten patients did not have viral load measured before censoring due to early death (8 subjects), retransplantation (1) and high dose steroid treatment for rejection (1).

Ninety-six patients (85%) had DNAemia detected. Twenty-five (22%) asymptomatic subjects received preemptive therapy, 21 (19%) of them had DNAemia ≥ 4000 UI/ml, 4 had therapy started at lower viral loads by their treating physician. None of them went on to develop CMV disease, nor did the 71 untreated patients with DNAemia < 4000 UI/ml.

The median time from transplant to a viral load ≥ 4000 UI/ml was 41 days (range 17–60). No first episode of DNAemia ≥ 4000 UI/ml was detected after day 60. Additional file 1: Fig. S1. The median time from treatment initiation to the first negative viral load was 27 days (range 16–126). Only one patient had recurrence of DNAemia ≥ 4000 UI/ml detected at month 6.

**Fig. 1:**
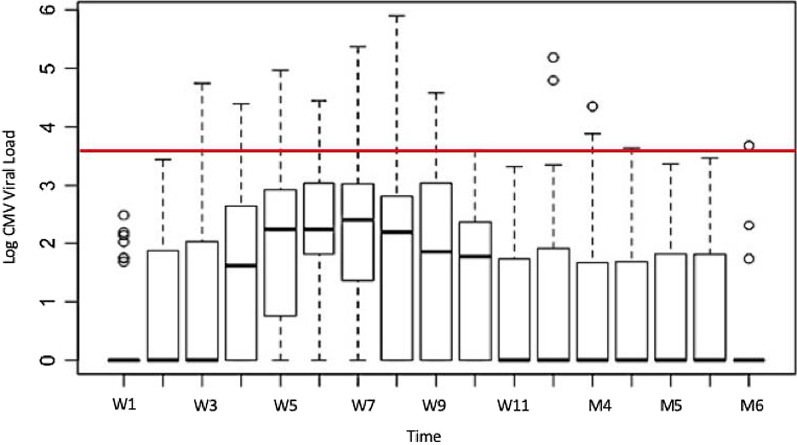
Graphical representation CMV viral loads over time. The red line represents the cut-off for treatment initiation (3.6 log, 4000 UI/ml). *W* week, *M* month

Figure 1 depicts viral load over time. The mixed lineal analysis reported a p value of < 0.0001, indicating that at least one mean differed from the rest. Difference between periods was explored further by comparing the means of month 1 vs. month 2 (p < 0.0001) and month 2 vs. month 3 (p < 0.0001).

Adherence to the centre’s protocol was stronger in the first posttransplant weeks. The number of viral loads measured decreased over time from > 90% of recommended determinations measured in the 1st week to around 50% of recommended determinations on month 6. Additional file 2: Table S1. The 1st year of the study had the lowest adherence to the number of recommended determinations, only a median of 30% of recommended determinations/patient were measured. Adherence improved with time, during the 3rd study year a median of 66% of recommended determinations/patient were documented.

Viral replication kinetics were calculated for 70 subjects. Table 2 depicts the parameters for the whole population, subjects who developed who developed DNAemia ≥ 4000 UI/ml and those who did not. Viral load growth rate was faster, doubling time was shorter and R_0_ was higher in patients who developed DNAemia ≥ 4000 UI/ml.

**Table 2:** Viral replication kinetics

Parameter	N = 70	CV ≥ 4000 N = 19	CV < 4000 N = 51	p
Growth rate (r) UI/ml/day	0.16 (0.07–0.33)	0.23 (0.16–0.5)	0.12 (0.02–0.26)	0.001
Duplication time (days)	3.75 (1.85–5.55)	2.91 (1.36–4.32)	4.23 (2.04–9.55)	0.026
R_0_ (basic reproductive number)	1.23 (1.1–1.48)	1.34 (1.23–1.73)	1.18 (1.04–1.37)	0.001

Data are presented as medians and interquartile range

Viral load growth rate was associated with development of DNAemia ≥ 4000 UI/ml by bivariate analysis as shown in Table 3. Viral load growth rate and autoimmune hepatitis were associated with development of DNAemia ≥ 4000 UI/ml on multivariate analysis (OR 7.35, CI 1.8–29.9 and OR 6.9, CI 1.3–35.5, respectively). Variables known to be associated with non-viral posttransplant infections (surgical time, transfusions, acute kidney injury, severity of liver disease) were not associated with development of DNAemia ≥ 4000 UI/ml [7] (Additional file 3: Table S2).

**Table 3:** Bivariate analysis to determine variables associated with development of DNAemia ≥ 4000UI/ml

Variable	Total N = 113	VL ≥ 4000 UI/ml N = 23	VL < 4000 UI/ml N = 90	OR (CI95%)	p
Age	52 (41–59)	55 (39–62)	51 (41–58)	1.01 (0.97–1.06)	0.34
Female sex	57 (50%)	13 (56%)	44 (49%)	1.35 (0.54.3.41)	0.51
Hepatitis C	23 (20%)	3 (9%)	10 (22%)	0.52 (0.14–1.94)	0.33
Cryptogenic cirrhosis	24 (21%)	3(13%)	21 (23%)	0.49 (0.13–1.82)	0.28
Autoimmune hepatitis	20 (18%)	7 (30%)	13 (14%)	2.58 (0.89–7.51)	0.08
Hepatocellular carcinoma	25 (22%)	5 (22%)	20 (22%)	0.97 (0.32–2.94)	0.96
Child–Pugh C	62 (55%)	12 (52%)	50 (55%)	0.87 (0.34–2.18)	0.77
MELD	35 (31%)	7 (30%)	28 (31%)	0.96 (0.35–2.61)	0.95
Donor CMV IgG positive	72/83 (87%)	18/19 (95%)	54/64 (84%)	3.33 (0.39–27.87)	0.26
Surgical time > 7 h	27/109 (25%)	4/22 (18%)	23/87 (26%)	0.61 (0.18–2.01)	0.42
> 6 blood cell transfused	24 (21%)	5 (22%)	19 (21%)	1.03 (0.34–3.15)	0.94
Reoperation	10 (9%)	1 (4%)	9 (10%)	0.4 (0.49–3.4)	0.4
Grade 3 AKI	25 (22%)	4 (17%)	21 (23%)	0.69 (0.21–2.25)	0.54
Posttransplant bacterial infection	27 (24%)	5 (22%)	22 (24%)	0.85 (0.28–2.58)	0.78
Viral load growth rate > 0.16 UI/ml/day	37/70 (53%)	15/19 (79%)	22/51 (43%)	4.94 (1.43–16.98)	0.011

Continuous variables are presented as medians and inter-quartile range. Categorical variables are presents as percentages

*VL* viral load, *MELD* Model for End Stage Liver Disease, *AKI* acute kidney injury

## Discussion

Cumulative incidence of CMV disease was 3.2% in this cohort. Asymptomatic DNAemia ≥ 4000 UI/ml was detected in 19%. Another cohort reported an incidence of CMV syndrome of 0.9% for D−/R+ and 7.1% for D+/R+. Nine percent of D−/R+ and 24% of D+/R+ received preemptive treatment for viremia [4]. Our reported incidence falls in the low end of the spectrum considering the fact that most donors were CMV IgG+. The positive predictive value of CMV viral load for CMV disease is not perfect. Despite this issue, viral load kinetics have been recommended as surrogate endpoints of CMV disease for research purposes [5, 9, 10]. The 19% incidence of DNAemia ≥ 4000 UI/ml falls within the expected range of CMV disease for this patient population if no preventive strategy is used [2]. These patients did not develop CMV disease, it can be concluded that the described strategy works well.

The number of viral load determinations decreased over time. There was low adherence during the 1st study year. The surveillance protocol had just been implemented and there were major disruptions in workflow secondary to the earthquake that struck Mexico City in 2017.

The median viral load doubling time of 2.9 days in patients who developed DNAemia ≥ 4000 UI/ml warrants strict monitoring. Relaxation of the monitoring strategy should be considered dangerous, particularly in the first 3 posttransplant months as most of the burden of CMV replication was concentrated in this period. No first episode of DNAemia ≥ 4000 UI/ml occurred after day 60. Late DNAemia ≥ 4000 UI/ml occurred only in 1 patient with previously treated DNAemia. Monitoring after the first 3 posttransplant months may not be necessary in patients with persistently negative DNAemia. Preemptive therapy is logistically challenging. Novel platforms of viral load measurement such as dried-blood spots (DBS) have the potential to ease the burden of monitoring for patients and transplant centres [11].

Viral load doubling time for patients who developed DNAemia > 4000 UI/ml was 2.9 days and the basic reproductive number was estimated to be 1.34. These parameters are consistent with those calculated in other cohorts [4]. Not unexpectedly, patients with DNAemia ≥ 4000 UI/ml had a faster viral load growth rate, shorter duplication time and higher R_0_ compared to those with DNAemia < 4000 UI/ml. Integrating replication kinetics into preemptive therapy algorithms is difficult. There is a wide overlap in parameters between patients who require preemptive therapy and those who do not. From a practical stance, we would recommend repeating the viral load measurement in 48–72 h if a worrisome elevation of DNAemia is noted.

Viral load growth rate and autoimmune hepatitis were significantly associated with development of DNAemia ≥ 4000 UI/ml. Patients with autoimmune hepatitis have a larger burden of immunosuppression. They are treated with glucocorticoids and immunosuppressants before the transplant. This might place them at a higher risk of CMV disease.

The main limitations of this study were its single-centre nature and the lack of CMV IgG status determination in all donors, this precluded finer analysis by donor CMV status. The major strength of this study is the population’s homogeneous intensity of immunosuppression. This makes the results applicable to similar populations bearing in mind that the viral load was measured in whole blood. Had the viral load been measured in plasma the threshold for initiation of preemptive therapy would have been lower. Administration of high-dose glucocorticoids for the treatment of rejection and retransplantation have been associated with a higher risk of CMV disease [12, 13]. Patients in these scenarios were excluded from this study and should have strict monitoring. Initiation of therapy should be considered at lower thresholds.

In conclusion, the incidence of CMV disease in seropositive liver transplant recipients is low when using preemptive therapy. Though risk is highest the first 3 posttransplant months we did document DNAemia as far as month 6. This represents a logistical challenge as it is difficult to maintain testing intensity that far after the transplant procedure. Despite the challenges of preemptive therapy, when done adequately, it results in a significant reduction of end-organ CMV disease. Understanding the CMV replication kinetics is important in designing these strategies but not practical to institute as routine clinical monitoring.

## Supplementary Information

**Additional file 1: Figure S1.** Kaplan–Meier curve depicting the development of CMV DNAemia > 4000 UI/ml over time in CMV seropositive recipients after liver transplantation.

**Additional file 2: Table S1.** Percentage of subjects with viral load determined.

**Additional file 3: Table S2.** Multivariate analysis to determine variables associated with development of viremia > 4000 UI/ml.

## Data Availability

The datasets used and/or analyzed during the current study are available from the corresponding author on reasonable request.

## Abbreviations

CMV: Cytomegalovirus
DBS: Dried blood spots
LTR: Liver transplant recipients
MEIA: Major immediate early antigen
MELD: Model for End Stage Liver Disease
r: Viral load growth rate
R_0_: Basic reproductive number
SOT: Solid organ transplant
Td: Vial load doubling time
WHO: World Health Organization
