# Supplementary material for: Incidence of Cytomegalovirus disease and viral replication kinetics in seropositive liver transplant recipients managed under preemptive therapy in a tertiary-care center in Mexico City: a retrospective cohort study
Source: BMC Infect Dis. 2022 Feb 14;22:155. doi: 10.1186/s12879-022-07123-w (PMC8845382; doi:10.1186/s12879-022-07123-w)
Supplement: Supplementary file 1 — Additional file 1: Figure S1. Kaplan–Meier curve depicting the development of CMV DNAemia > 4000 UI/ml over time in CMV seropositive recipients after liver transplantation. [file 12879_2022_7123_MOESM1_ESM.pdf]

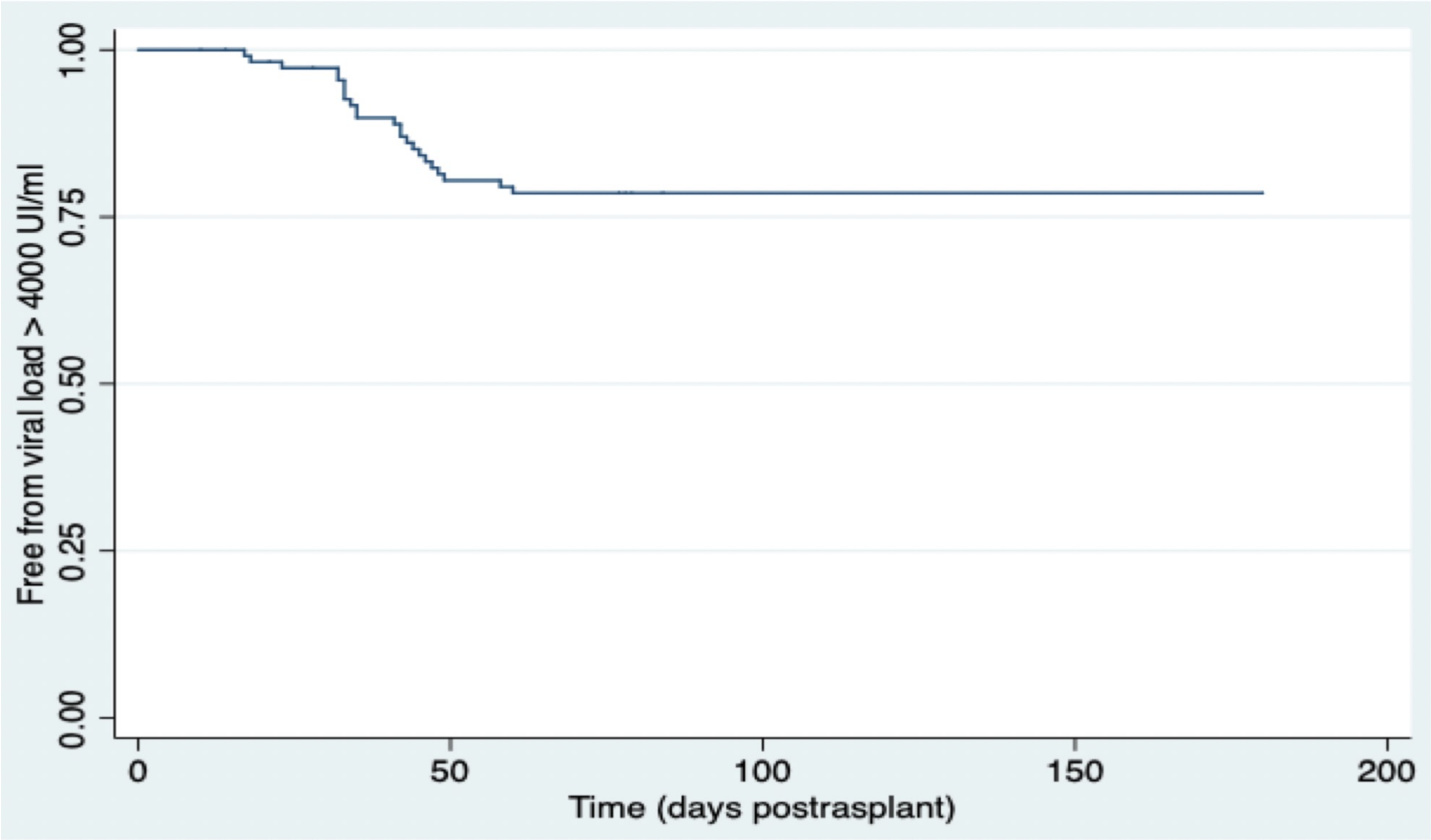

Additional Figure S1. Kaplan-Meier curve depicting the development of CMV DNAemia >4000 UI/ml over time in CMV seropositive recipients after liver transplantation.
