# Supplementary material for: Incidence of Cytomegalovirus disease and viral replication kinetics in seropositive liver transplant recipients managed under preemptive therapy in a tertiary-care center in Mexico City: a retrospective cohort study
Source: BMC Infect Dis. 2022 Feb 14;22:155. doi: 10.1186/s12879-022-07123-w (PMC8845382; doi:10.1186/s12879-022-07123-w)
Supplement: Supplementary file 2 — Additional file 2: Table S1. Percentage of subjects with viral load determined. [file 12879_2022_7123_MOESM2_ESM.pdf]

Time	W1	W2	W3	W4	W5	W6	W7	W8	W9	W10	Wk11	Wk12	M4	M4.5	M5	M5.5	M6
Population	113	113	112	111	110	109	108	108	107	107	107	106	105	103	103	103	103
Percent of measurements	91	83	65	65	68	56	58	56	75	50	53	44	58	54	52	36	49

Additional Table S1: Percentage of subjects with viral load determined. The population progressively decreased due to censoring for death, retransplantation or treatment of rejection.
W: Week, M: Month
