# Supplementary material for: Incidence of Cytomegalovirus disease and viral replication kinetics in seropositive liver transplant recipients managed under preemptive therapy in a tertiary-care center in Mexico City: a retrospective cohort study
Source: BMC Infect Dis. 2022 Feb 14;22:155. doi: 10.1186/s12879-022-07123-w (PMC8845382; doi:10.1186/s12879-022-07123-w)
Supplement: Supplementary file 3 — Additional file 3: Table S2. Multivariate analysis to determine variables associated with development of viremia > 4000 UI/ml. [file 12879_2022_7123_MOESM3_ESM.pdf]

Variable	OR (IC95%)	p
Age	1.04 (0.98-1.1)	0.15
Autoimmune hepatitis	6.93 (1.35-35.51)	0.02
MELD>20	0.63 (0.17-2.3)	0.49
Surgical time >7 hours	0.86 (0.17-4.17)	0.85
>6 blood units transfused	0.69 (0.13-3.6)	0.66
Grade 3 AKI	0.48 (0.09-2.40)	0.37
Viral load growth rate>0.16	7.35 (1.8-29.91)	0.005

Additional Table S2. Multivariate analysis to determine variables associated with development of viremia >4000 UI/ml. MELD: Model for End-Stage Liver Disease. AKI: Acute Kidney Injury.
